# Investigation of 3-dimensional radiographic imaging as treated with Hyu Spinal corrective technique

**DOI:** 10.1186/1748-7161-8-S2-O17

**Published:** 2013-09-18

**Authors:** Kim HoSeong, Noh Dong Koog, Koh Jae-Hyun, Kim Donghyun, Kim JunLae

**Affiliations:** 1 Department of Physical Medicine and Rehabilitation, Seoul Hyu Clinic, Gyeonggi-do, Korea

## Background

Adolescent idiopathic scoliosis (AIS) is a common, costly, and progressive spinal deformity that affects 3-dimensional (3D) neuromuscular control of the axial spinal musculature. Conventional studies of spinal corrective techniques have shown improvements in cardiopulmonary function, strength, mobility, pain and body image. Nevertheless, the majority of AIS studies predominantly focus on alleviating 1- or 2-dimensional (2D) spinal deformity (frontal or sagittal) risk factors, and have not specifically targeted multi-dimensional risk factors associated with AIS.

## Purpose

The goal of this study was to compare the 3D change of spine and pelvic alignment in AIS patients after applying a Hyu Spinal Technique (HST; focusing on 3D-correction along with dynamic lumbo-pelvic and trunk stabilization) and Conventional Exercise (CE).

## Methods

Idiopathic scoliosis (N = 62,13 males) between 10 and 19 years of age (14.23 ±2.31 years) were treated either with the HST or CE in outpatient sessions lasting approximately one hour each, 2-3 times a week. A diagnostic 3D X ray imaging technique was used to determine intervention-related changes in the Cobb angle, thoracic kyphosis angle, lumbar lordosis (LL) angle, pelvic incidence (PI) and vertebral rotation (VR, Nash-Moe method). The SRS 22 post-intervention survey was used. Data were analyzed using the non-parametric Mann-Whitney U-test and Wilcoxon signed rank test at p < 0.05.

## Results

When compared to CE, the HST showed greater improvements in Cobb angle (p = 0.014), LL angle (p = 0.010), PI (p = 0.010), VR (p = 0.043) and SRS 22 scores (self-image and treatment satisfaction subscale scores and total score, p = 0.026, p = 0.039 and p =0.041 respectively). There were no significant changes in the other measures between the two groups.

## Conclusions and discussion

The results indicate that the HST as 3D spinal corrective technique is effective for correcting spinal malalignment not only in the frontal plane, but in the sagittal and transverse planes. This is the first study using advanced radiographic imaging to investigate the effects of a 3D spinal corrective technique on spinal curvatures and self-image in AIS; thus providing important clinical rationale for the 3Dapproach in the effective management of AIS.
